# Transcriptomic insights into the allelopathic effects of the garlic allelochemical diallyl disulfide on tomato roots

**DOI:** 10.1038/srep38902

**Published:** 2016-12-12

**Authors:** Fang Cheng, Zhi-Hui Cheng, Huan-Wen Meng

**Affiliations:** 1 College of Horticulture, Northwest A&F University, Taicheng Road No. 3,Yangling, Shaanxi 712100 China

## Abstract

Garlic is an allelopathic crop that can alleviate the obstacles to continuous cropping of vegetable crops. Diallyl disulfide (DADS), one of the most important allelochemicals in garlic, promotes tomato root growth. Therefore, the global transcriptome profiles of DADS-treated tomato roots over time were investigated to reveal the potential growth-promoting mechanisms. We detected 1828, 1296 and 1190 differentially expressed genes (DEGs) in the 4, 24 and 48 h samples, respectively. Most DEGs involved in assimilatory sulfate reduction and glutathione metabolism were up-regulated after short-term (4 h) DADS treatment. In addition, increased activity of defensive enzymes and up-regulation of six *peroxidase* genes were observed, suggesting that DADS could induce tomato resistance. In plant-pathogen interactions, DEGs related to calcium signaling were primarily inhibited, while those encoding pathogenesis-related proteins were primarily up-regulated. Although plant hormone synthesis and signal transduction were both significantly affected by DADS, the expression trends of the genes in these two pathways were conflicting. This research provides comprehensive information concerning the changes in the tomato root transcriptome affected by DADS and may help direct further studies on DADS-responsive genes to enhance the current understanding of the mechanisms by which DADS alleviates the obstacles to continuous cropping.

Garlic (*Allium sativum* L.) is a traditional vegetable and popular garden crop in China. Garlic has been recognized as a good preceding crop in agriculture production for its beneficial allelopathic effects on alleviating the obstacles to continuous cropping, such as enhancing soil fertility by changing nutrient levels, soil enzymatic activity and microbial community composition, alleviating soil sickness, and improving crop quality and yield^1^,^2^,^3^,^4^,^5^,^6^. A previous study reported that plant chlorophyll content, photosynthetic rate and antioxidant enzyme activities could also be improved through intercropping with garlic^7^.

Diallyl disulfide (DADS) is a volatile organosulfur compound found in garlic and other Allium plants^8^. It was reported that DADS could reduce the incidence rate of cancers and inhibit the proliferation of cancer cells. The anticancer mechanisms of DADS include the activation of enzymes that can detoxify carcinogens; suppression of DNA adduct formation; antioxidation; cell-cycle regulation; induction of apoptosis and differentiation; histone modification; and inhibition of blood vessel formation and invasion^9^,^10^. Demeule *et al*.^11^ showed that DADS could increase glutathione transferase (GST) activity, glutathione (GSH) levels and the expression of *Mrp2*, which mediates the transport of GSH-conjugates in male Sprague-Dawley rats^11^. Studies of the nonenzymatic antioxidant activity of DADS have shown that this compound is a potent agent to enhance lipid stability^12^. DADS can also induce cell cycle arrest in the G_2_/M phase and increase the expression of cyclin B1 in human colon cancer HCT-116 cells^13^. Using transcriptomics and proteomics, researchers have demonstrated that DADS-affected genes and proteins in cancer cells are involved in signal transduction, cell proliferation and differentiation, apoptosis, cell cycle regulation, DNA replication and transcription, protein degradation, immunity, the cytoskeleton, oxidation-reduction and metabolism^14^,^15^.

DADS is one of the most important allelochemicals in garlic root exudates and garlic straw aqueous extracts^16^,^17^,^18^. In a previous study, we demonstrated that DADS exerts a biphasic, dose-dependent effect on tomato primary root elongation; DADS promoted tomato root elongation at low concentrations (0.01–0.62 mM) but inhibited elongation at high concentrations (6.2–20.67)^19^. Based on previous studies, we propose that DADS may also play an important role in alleviating the obstacles to continuous cropping of vegetables.

In recent years, substantial transcriptomics data have been used to characterize the genes governing the key developmental and metabolic processes in tomato^20^. However, so far, the DADS-regulated and DADS-responsive mechanisms in tomato have not been studied in detail. In the present study, the dynamic transcriptome and activity of defense enzymes in tomato roots treated with DADS were investigated. Root samples from a time course of 0, 4, 24 and 48 h following DADS treatment were detected to identify the major genes and pathways involved in DADS activation. The present study represents the first transcriptome sequencing assessment of the effects of DADS on tomato, which not only will highlight the mechanisms of DADS in plants but also provide important information for further studies of DADS-responsive genes and for research using DADS to alleviate continuous cropping obstacle of vegetables.

## Results

### RNA sequencing results and quality assessment

In the present study, 12 differentially expressed gene (DEG) libraries were constructed from tomato roots treated with DADS for 0, 4, 24 and 48 h to identify DEGs in response to DADS. High quality (HQ) base reads were obtained in the present study (Supplementary Fig. S1): more than 99% of the raw reads passed filtration. The quality and mapping results for all filtered base counts and reads are listed in Supplementary Tables S1 and S2. The Q30 scores of the clean bases reached over 93% in all samples, and the mapped ratio of the samples reached at least 89% after rRNA was removed. In addition, sequencing saturation analysis revealed that when the number of clean reads reached approximately 15 M, the number of identified genes did not increase (Supplementary Fig. S2). The correlation between replicates was also calculated to evaluate the reliability of the results. Pearson’s correlation, a value of nearly 1, indicates the significant repeatability of the experiments. The results of the present study indicated that all Pearson correlations were higher than 0.95 (Supplementary Fig. S3).

### DEGs among all samples

**Figure 1:**
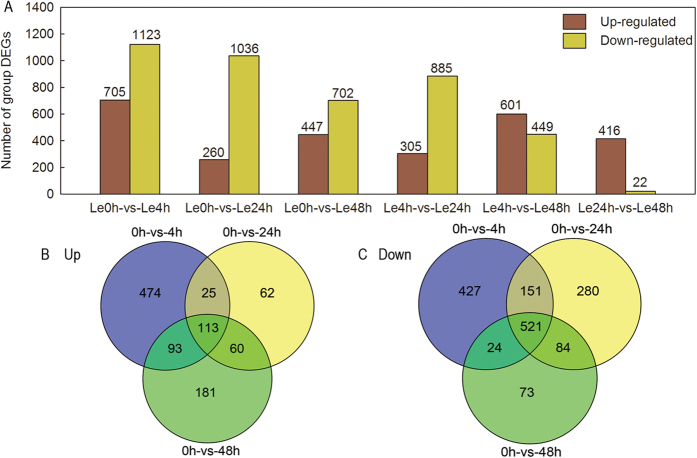
The number of DEGs among all samples after DADS treatment. (**A**) the number of up/down-regulated genes compared between different lengths of DADS treatment. (**B**,**C**) Venn diagram for DEGs identified at each time point after DADS application. The threshold of FDR ≤ 0.05 and log_2_|FC (ratio of DADS/control)| ≥ 1 were used to judge the significant differences in gene expression.

The expression level of each gene was calculated based on the number of fragments uniquely mapped to a specific gene and the total number of uniquely mapped fragments in the sample. The detailed information on the DEGs for all examples is summarized in Supplementary Table S3. The statistics of the DEGs are shown in Fig. 1; a total of 2568 DEGs were considered differentially expressed between control and DADS-treated tomato roots. Specifically, 705 genes were up-regulated and 1123 genes were down-regulated in the 4 h sample, 260 genes were up-regulated and 1036 genes were down-regulated in the 24 h sample, and 305 genes were up-regulated and 885 genes were down-regulated in the 48 h sample (Fig. 1A). Among the 634 DEGs co-regulated during DADS treatment, 113 genes were commonly up-regulated (Fig. 1B), and 521 genes were commonly down-regulated (Fig. 1C).

### Gene ontology classification of DEGs

To determine the functions of the DEGs in response to DADS, the significant DEGs were classified into three categories using GO-term analysis. In ‘biological process’, 13 GO terms were significantly enriched in the 4 h DADS-treated tomato roots, among which ‘oxidation-reduction process’, ‘response to ethylene’, and ‘sulfate reduction’ were highly enriched (see Spplementary Table S4). There were many more GO terms enriched in ‘biological process’ in the 24 h DADS-treated tomato roots than at 4 h. In addition to ‘ethylene-activated signaling pathway’, GO terms such as ‘salicylic acid mediated signaling pathway’, ‘hormone-mediated signaling pathway’ and ‘response to hormone’ were significantly enriched, suggesting that plant hormone synthesis and signal transduction can be influenced through DADS. Interestingly, we observed that the defense responsive categories ‘immune system process’, and ‘regulation of defense response’ were enriched in response to DADS, suggesting that DADS may change the resistance abilities of tomato. Lastly, in the 48 h sample, categories related to response to plant hormones, signal transduction and defense were also enriched in GO analysis. Taken together, plant hormone synthesis and signal transduction, and defense response in tomato root were influenced by DADS.

### KEGG pathway enrichment analysis of DEGs

To investigate the biological pathways induced by DADS in tomato roots, the identified DEGs were further mapped to reference pathways in the KEGG PATHWAY Database (http://www.genome.jp/kegg/pathway.html). As shown in Table 1, ‘glutathione metabolism pathway’ was the most significant pathway among the primary DADS-responsive genes. Two enriched pathways, ‘plant hormone signal transduction’ and ‘plant-pathogen interaction’, were both notably changed during DADS treatment (4, 24 and 48 h), suggesting that genes related to these pathways are involved in the DADS response of tomato roots. In addition, ‘glutathione metabolism pathway’ and ‘cysteine (Cys) and methionine metabolism’ were also significantly enriched after 4 h of DADS treatment in tomato roots. These two pathways are related to sulfur metabolism, suggesting that DADS may affect sulfur assimilation and transformation.

### DEGs and related enzymes involved in assimilatory sulfate reduction and GSH metabolism

**Figure 2:**
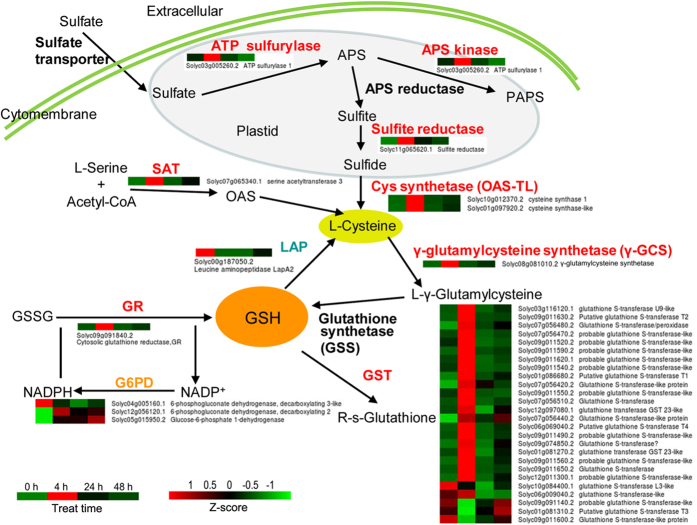
DEGs involved in assimilatory sulfate reduction and the GSH metabolism pathway in DADS-treated tomato roots. APS: adenosine-5′-phosphosulfate; SAT: L-serine acetyltransferase; PAPS: 3′-phosphoadenosine-5′-phosphosulfate; OAS-TL: cysteine (Cys) synthase; γ-GCS: γ-glutamylcysteine synthetase; OAS: O-acetylserine; GSS: glutathione synthetase; GR: glutathione reductase; G6PD: glucose-6-phosphate 1-dehydrogenase; GSSG: glutathione; GST: glutathione transferase; LAP: leucine aminopeptidase. Relative expression levels were normalized based on the Z-score and are shown on a color gradient from low (green) to high (red).

**Figure 3:**
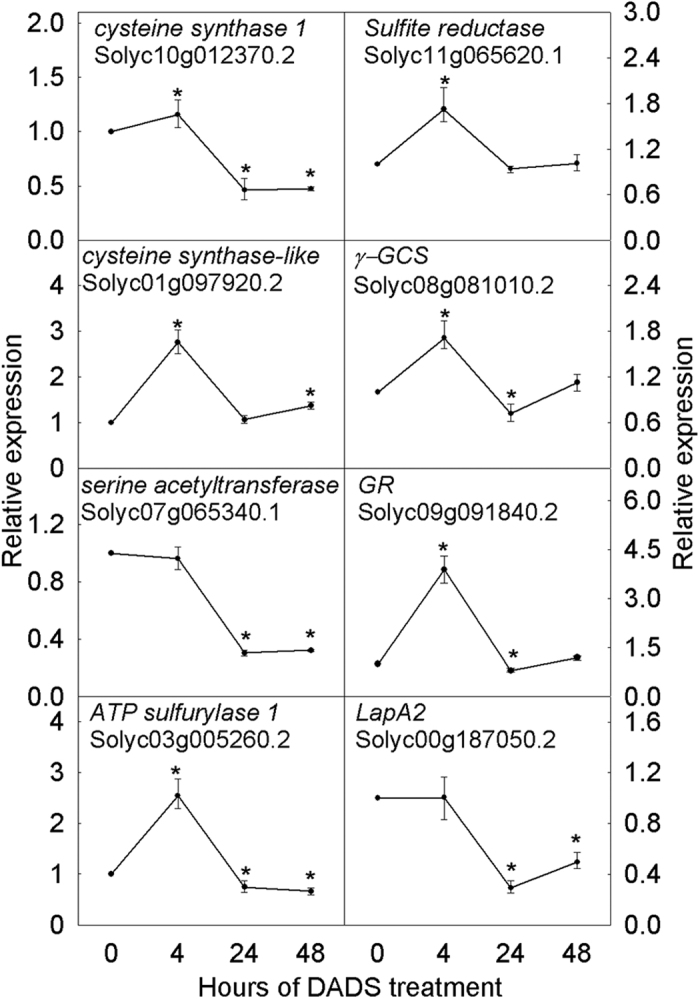
Effects of DADS on the expressions of genes related to assimilatory sulfate reduction and GSH biosynthesis. The roots were sampled 4, 24 and 48 h after DADS treatment. Roots without DADS treatment (0 h) were used as controls. The expression levels were determined using qRT-PCR and are expressed as the means ± SE. * Represents a significant difference between DADS treatment and control by Student’s t-test (P < 0.05), n = 3.

**Figure 4:**
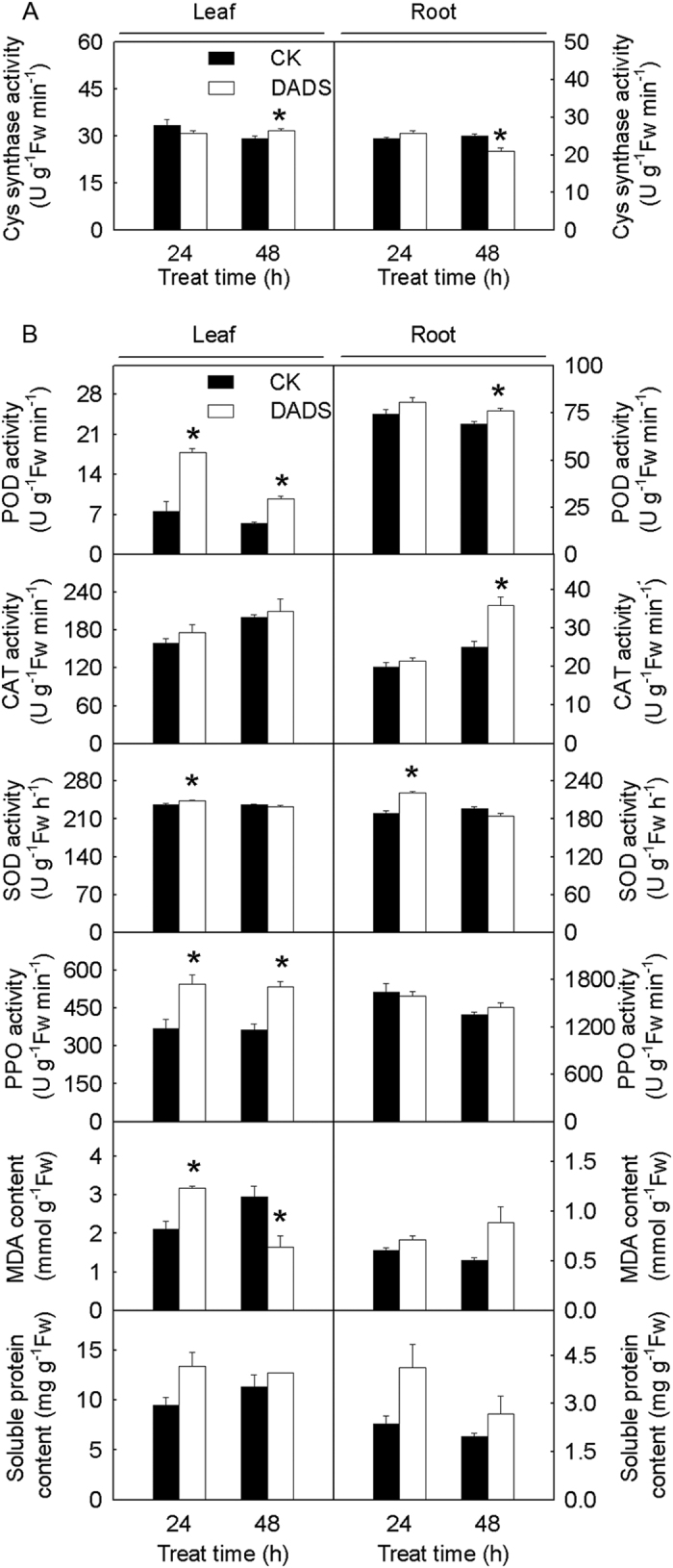
Effects of DADS on tomato Cys synthase, defense enzyme activities and soluble protein and MDA content. (**A**) the effects of DADS on tomato Cys synthase activity. (**B**) the influences of DADS on tomato defense enzymes (POD, CAT, SOD, and PPO) activities and soluble protein and MDA content. Tomato plants at the 3-leaf stage were treated with 0.21 mM DADS. The left-Y-axes represent leaf physiological indices and the right-Y-axes represent root physiological indices. The scales of the left- and right-Y-axes are different. The data for a particular treatment are presented as the means ± SE. *Significant differences according to Student’s t-test (P < 0.05), n = 3.

According to the KEGG pathway enrichment analysis and gene function annotation, we observed that most of the DEGs related to assimilatory sulfate reduction and GSH metabolism were induced by DADS (Figs 2 and 3). In assimilatory sulfate reduction, the expression of one gene (Solyc03g005260.2) encoding ATP sulfurylase and APS (adenosine-5′-phosphosulfate) kinase, one gene (Solyc11g065620.1 *sir*) encoding sulfite reductase, one gene (Solyc07g065340.1) encoding L-serine acetyltransferase (SAT) and two genes (Solyc10g012370.2 and Solyc01g097920.2) encoding Cys synthase (OAS-TL) were significantly up-regulated after short-term (4 h) DADS treatment (Fig. 2). However, only the gene encoding SAT was decreased during DADS treatment, as shown by qRT-PCR (Fig. 3). Similarly, during GSH synthesis, one gene (Solyc08g081010.2) encoding γ-glutamylcysteine synthetase (γ-GCS) and one gene (Solyc09g091840.2) encoding cytosolic glutathione reductase (GR) were significantly induced after DADS treatment for 4 h (Fig. 2). Two of three genes encoding glucose-6-phosphate 1-dehydrogenase (G6PD), which is involved in the transformation of oxidized glutathione (GSSG) to GSH, were also increased after 4 h of DADS treatment. In GSH degradation, the expression of one gene (Solyc00g187050.2) encoding leucine aminopeptidase (LAP) was consistently down-regulated in response to DADS treatment, although the suppression efficiency was gradually weakened. The same result was also obtained using qRT-PCR. In GSH metabolism, 26 genes encoding GST were specifically identified, of which, 13 GST genes were induced at 4 h after DADS treatment. In addition, the expression of eight other *GST* genes was higher during DADS application than in the control. The transcriptional regulation of the genes related to GSH biosynthesis and catabolism suggests increased content of GSH and activation of GST by DADS. Cys synthase is a key enzyme associated with sulfur assimilation and GSH metabolism. The effects of DADS on Cys synthase activity are shown in Fig. 4A. The Cys synthase activity in tomato root was slightly increased after 24 h but significantly decreased after 48 h. However, Cys synthase activity in tomato leaf was opposite of that in the root after DADS treatment.

### DEGs involved in oxidation resistance

The effects of DADS on the activity of tomato root defense enzymes [e.g., superoxide dismutase (SOD), peroxidase (POD), catalase (CAT) and polyphenol oxidase (PPO)] and on the contents of soluble protein and malonaldehyde (MDA) are shown in Fig. 4B. The activity of POD, CAT, SOD and PPO in tomato leaf and root were increased after DADS treatment for 24 and 48 h. DADS also increased the soluble protein contents in the tomato leaf and root. After DADS treatment for 24 h, the leaf MDA content increased but was significantly decreased after 48 h. The MDA contents in tomato root were increased after DADS treatment but showed no significant difference. In transcriptome analysis, several DEGs responsible for reactive oxygen species (ROS) scavenging were screened. Specifically, 24 *POD* genes and one *PPO* gene were identified (Supplementary Table S5). The relative expression of 13 *POD* genes increased at 4 h after DADS treatment, and the promotion effects of six *POD* genes lasted for 48 h. In contrast, the expression of nine *POD* genes was inhibited during DADS treatment. This result suggests that DADS could stimulate the antioxidant ability of tomato. Only one significantly affected *PPO* gene was detected in tomato root and was down-regulated at 4 h but up-regulated at 24 and 48 h after DADS application.

### DEGs enriched in plant-pathogen interaction pathway after DADS treatment

In the present study, several disease resistance-related genes were significantly enriched in plant-pathogen interaction pathway, according to KEGG analysis (Supplementary Table S6). Specifically, two of three respiratory burst oxidase homolog (*RBOH*) genes, all three cyclic nucleotide-gated ion channel (*CNGCs*) genes, 15 of 16 calcium-binding protein (*CaM*/*CML*) genes, the three jasmonate ZIM-domain (*JAZ*) genes and a basic helix-loop-helix transcription factor (*MYC2*) were down-regulated after DADS treatment at the three time points, suggesting that the expression of genes relevant to resistance was strongly inhibited in response to DADS, and the interactions between plants and pathogens are also primarily reduced in response to DADS. However, five of nine *PR* genes were consistently up-regulated in response to DADS. This finding revealed that *PR* genes may have significant functions in response to DADS application.

### Plant hormone synthesis in response to DADS treatment

In the present study, the expression of the genes involved in the biosynthesis of phytohormones, such as auxin, cytokinin (CTK), abscisic acid (ABA), gibberellin (GA) and ethylene, also showed significant changes after DADS treatment (Supplementary Table S7). In auxin biosynthesis, the gene (Solyc02g084640.2) encoding aldehyde dehydrogenase and another gene (Solyc09g074430.2) encoding flavin-containing monooxygenase were significantly down-regulated 4 h after DADS treatment. In CTK biosynthesis, the crucial enzyme adenylate isopentenyltransferase, encoded by Solyc09g064910.1, was up-regulated by DADS, but the promotion effect weakened after 48 h. The genes encoding CTK oxidase/dehydrogenase and glucosyltransferase had different expression patterns. This finding may indicate the dynamic control of the CTK content in response to DADS. In GA biosynthesis, the expression of the genes encoding gibberellin 20-oxidase-3 (Solyc11g072310.1) and 3b-hydroxylase (Solyc06g066820.2), which catalyze gibberellins from their inactive to active forms, was up-regulated from 4 to 48 h after DADS treatment. Almost all DEGs associated with ABA biosynthesis were inhibited through DADS. For example, the expression of the gene Solyc07g056570.1, which encodes nine-cis-epoxycarotenoid dioxygenase (NCED), the key rate-limiting enzyme in ABA synthesis, was down-regulated 1.97–2.81-fold after DADS treatment for 4 and 24 h compared with treatment at 0 h. In ethylene synthesis, except the gene Solyc01g095080.2, the genes encoding 1-aminocyclopropane-1-carboxylate synthase (ACS) were all inhibited in response to DADS, while the genes encoding 1-aminocyclopropane-1-carboxylate oxidase (ACO) showed different expression patterns. The apparent regulation of the genes involved in phytohormone synthesis and catabolism suggests that DADS has dynamic and multiple effects on phytohormone synthesis in tomato root.

### Plant hormone signal transduction pathways in response to DADS treatment

**Figure 5:**
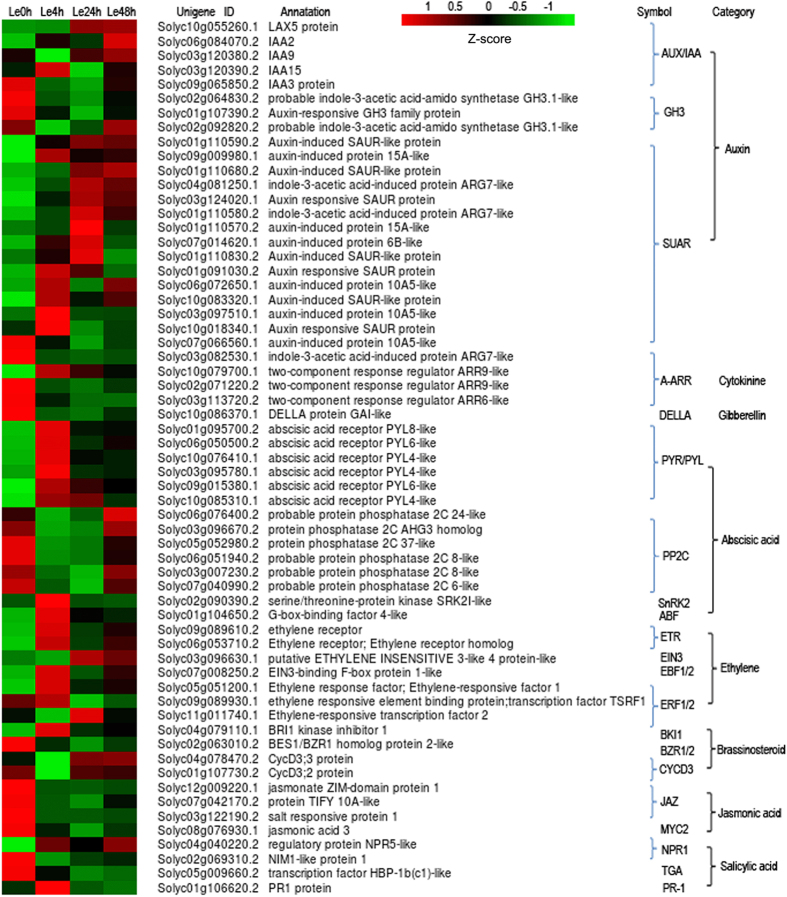
Heat map of transcriptional levels for DEGs enriched in plant hormone signal transduction pathways through KEGG analysis. In this heat map, the columns represent tomato root samples treated with DADS for different times, and the rows represent DEGs enriched in plant hormone signal transduction. Relative expression levels were normalized based on the Z-score and shown as a color gradient from low (green) to high (red).

The signal transduction pathways of plant hormones, such as auxin, CTK, GA, ABA, ethylene, jasmonic acid (JA) and salicylic acid (SA), were all affected by DADS. In the present study, 61 annotated DEGs were significantly enriched in the ‘plant hormone signal transduction’ pathway after DADS application (Fig. 5). In auxin signal transduction, the five genes belonging to the auxin/indoleacetic acid (Aux/IAA), which encode short-lived nuclear proteins, were differentially expressed, among which two genes (*IAA2* and *LAX5*) were up-regulated and one gene (*IAA3*) was down-regulated compared with the control, and all three DEGs encoding gretchenhagen-3 (GH3) were down-regulated. A total of 12 out of the 16 DEGs encoding small auxin-up RNAs (SAURs) in auxin signal transduction were up-regulated. This result indicates that auxin signal transduction is primarily up-regulated in response to DADS treatment. In the CTK signaling pathway, one of three genes encoding type-a response regulators (A-ARRs) was up-regulated, while the other two genes were down-regulated. Only one gene encoding a DELLA protein was significantly down-regulated in the GA signal transduction pathway. In ABA signal transduction, eight genes, including six *abscisic acid receptor* (*PYR*/*PYL*) genes, one *SNF1 related protein kinase 2* (*SnRK2*) gene and one *ABA responsive element binding factor* (*ABF*) gene, showed increased expression in response to DADS treatment, but the expression of six *type 2C protein phosphatase* (*PP2C*) genes was down-regulated. Genes involved in ethylene signaling were also affected in response to DADS. For example, four DEGs, including two *ethylene receptor* (*ETR*) genes, one *ethylene insensitive 3b* (*EIN3*) gene and one *ERF transcription factor* (*ERF1*/*2*) gene, showed up-regulated expression after DADS treatment, while the other three DEGs showed up and/down-regulation at different time points after DADS application. Taken together, DEGs in ABA and ethylene-signaling pathways were more up-regulated than down-regulated, suggesting that the genes associated with the signal transduction of these plant hormones were primarily induced in response to DADS treatment. In contrast, the DEGs in JA signaling transduction were all inhibited after DADS treatment.

### Quantitative real-time (qRT) PCR validation of DEGs

**Figure 6:**
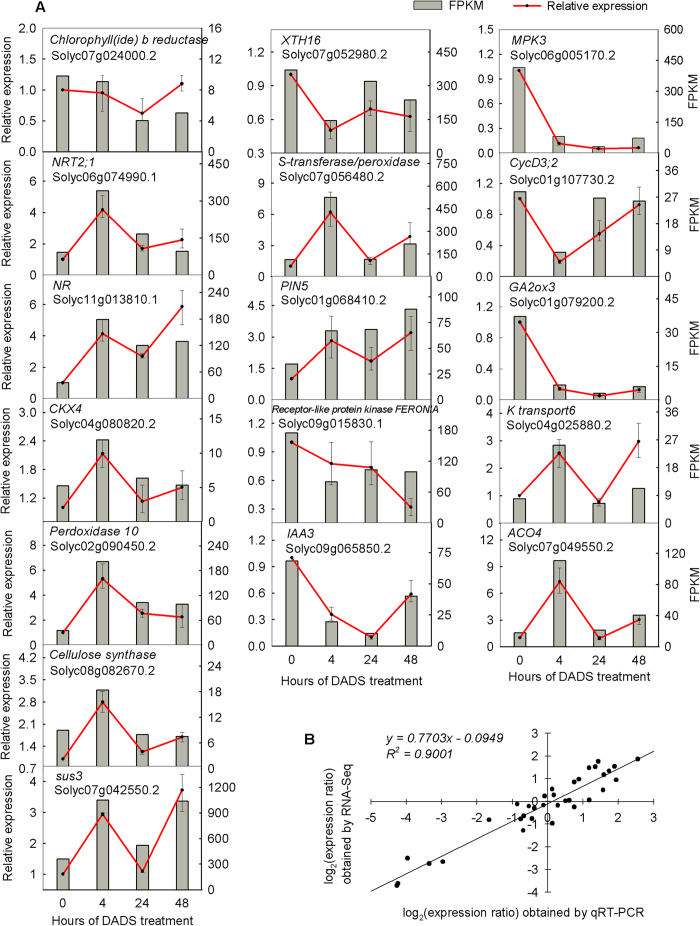
qRT-PCR analysis of DEGs in tomato root after DADS treatment. (**A**) expression levels revealed using qRT-PCR and RNA-seq of 17 randomly selected DEGs. The Y-axis on the left shows the relative gene expression levels analyzed using qRT-PCR (red lines) with 3 replicates, while the Y-axis on the right shows the corresponding expression data for RNA-seq (gray histogram). (**B**) Comparison between the log_2_ of expression ratios of DGEs obtained from RNA-seq and qRT-PCR.

The accuracy and reproducibility of RNA-seq data were confirmed by 17 randomly selected DEGs through qRT-PCR. The DEGs encode metabolism and signal transduction proteins, protein kinases, transferases, and oxidoreductases and showed up-regulated, down-regulated, and unaffected expression trends. The FPKM (fragment per kilobase of exon model per million mapped reads) and qRT-PCR results are shown in Fig. 6A. In addition, the correlation between the transcript abundance determined through qRT-PCR and RNA-seq was examined using linear regression analysis. The Pearson’s correlation coefficient reached 0.949 (Fig. 6B), and the results showed that the gene expression ratios between RNA-seq and qRT-PCR present a significantly positive correlation, indicating that the RNA-seq data in the present study are reliable.

## Discussion

### DADS activated assimilatory sulfate reduction and GSH metabolism

Sulfur is an essential macronutrient for plants and is also an important substrate in biotic and abiotic stress reactions^21^. After uptake, SO_4_^2−^ is reduced to APS by ATP sulfurylase, catalyzed to sulfite through APS reductase, and subsequently reduced to sulfide by sulfite reductase. The key enzyme of plant sulfur metabolism, Cys synthase, usually called O-acetylserine (thiol) lyase (OAS-TL), catalyzes the synthesis of Cys from sulfide ion (S^2−^) and O-acetylserine (OAS) in the last step of the photosynthetic assimilation of sulfate in plants^21^. Cys is the precursor of many important sulfur-containing compounds, which participate in plant defense systems^21^. In the present study, genes encoding SO_4_^2−^ transporters were not affected by DADS. However, the expression of genes related to sulfur assimilation, such as ATP sulfurylase, APS kinase, sulfite reductase, SAT and OAS-TL, were up-regulated 4 h after DADS treatment, suggesting that DADS promotes the assimilation of sulfur in plant cells and may increase the Cys content, thereby providing an abundant substrate for subsequent metabolism. In addition, the decreased activity of Cys synthase after 48 h may reflect the negative regulation of increased Cys pools induced by DADS in tomato root.

GSH plays a key role in plant tissue antioxidant defenses (e.g., quenching ROS, scavenging peroxides and free radicals) and in the signal transduction of redox-sensitive elements in organisms and is a major sulfur-containing substrate in antioxidative defense. The size of the GSH pool and its redox status are tightly correlated with the tolerance of plants to ROS, xenobiotics, abiotic and biotic stresses^22^. GSH is synthesized from glutamic acid, Cys and glycine through two ATP-dependent reactions catalyzed by γ-GCS and glutathione synthetase (GSS)^23^. In GSH metabolism, plant *GSTs* exhibit diverse expression patterns under biotic and abiotic stresses^24^. In the present study, the results of the KEGG analysis revealed that the ‘glutathione metabolism pathway’ is the most significant pathway induced early by DADS and is closely associated with DADS regulation in tomato root. The up-regulation of GSH synthesis genes (*γ-GCS* and *GR*), *GSTs* and the down-regulation of the GSH degradation gene (*LapA2*) suggest that DADS increased GSH content and GST activity and might improve the resistance of tomato root. Similarly, it has been reported that DADS could increase GST activity and GSH level and has a protective effect against human cancers^11^.

### DADS induced enzymatic antioxidant systems

Oxidative stress produces ROS, which causes membrane lipid peroxidation, leads to cellular function damage, and inhibits plant growth and development^25^. Plants have developed a complex ROS-scavenging system for protection from the damages resulting from oxidative stress. The antioxidant enzymes SOD, POD, and CAT, play crucial roles in scavenging ROS^26^. PPO catalyzes the oxidation of ortho-diphenols to ortho-quinones using molecular oxygen and causes typical browning reactions following tissue damage^27^. Hence, PPO can affect local levels of oxygen and ROS and may be important in plant defense^28^. In a previous study, Ahmad *et al*. (2013) showed that the activities of CAT and POD in pepper were significantly enhanced when intercropping with garlic, while that of PPO was inhibited^7^. In addition, the activities of SOD, POD and CAT in cucumber (*Cucumis sativus*) leaves were also increased in response to aqueous garlic extracts at proper concentrations^29^. Wang *et al*. inferred that the effect of garlic on alleviating the obstacles to continuous cropping may partly reflect the stimulation of defense enzymes^30^. Similar results were observed in the present study. Moreover, the up-regulation of six *PODs* during DADS treatment suggests that these genes play a major role in scavenging active oxygen, and DADS could induce and enhance the clearance of ROS, thereby increasing tomato resistance.

Plants undergo oxidative stress in response to higher levels of ROS. However, at low levels, ROS are also known as secondary messengers that trigger tolerance to various abiotic and biotic stresses^31^. Hayat *et al*. showed that aqueous garlic extract altered the defense mechanism of cucumber seedlings through the activation of ROS at mild concentrations^29^. However, in the present study, the ROS content may decrease in response to the down-regulation of the *RBOH* genes involved in ROS production (Supplementary Table S6), suggesting that ROS may not be the active signal in inducing tomato resistance under 0.21 mM DADS conditions.

DADS slowly releases hydrogen sulfide (H_2_S), leading to slight H_2_S accumulation in cells^32^. H_2_S regulates plant growth and improves plant antioxidant systems, including GSH content, SOD, POD, CAT and GR activities and their related genes, to alleviate damages caused by numerous abiotic stresses^33^. Sulfite reductase reduces sulfite into sulfide, such as H_2_S. In the present study, the expression of *sir*, which encodes sulfite reductase, was induced in response to DADS treatment after 4 h (Figs 2 and 3). This finding suggests that the H_2_S level in tomato may also increase in response to DADS. Moreover, H_2_S may play a considerable role in the activation of tomato antioxidant systems. However, the direct or indirect relationship of DADS with H_2_S in tomato induced resistance and whether the H_2_S was released by DADS or induced by DADS requires further study.

### DADS inhibited plant PTI but induced ETI

The inducible plant defense responses to pathogens primarily include two stages^34^. In the first stage, plant pattern recognition receptors recognize the presence of pathogen-associated molecular patterns (PAMPs), eliciting PAMP-triggered immunity (PTI)^35^. The second stage is elicited by the recognition of effectors secreted from pathogens, eventually leading to effector-triggered immunity (ETI)^36^. Calcium (Ca^2+^) signaling is essential in the Ca^2+^-calmodulin (CaM) signal system, which regulates gene expression and stress tolerance and plays a vital role in both PTI and ETI responses^37^. Calcium sensor proteins, including calcium-dependent protein kinase (CDPK), calcium-related proteins, CaM and calmodulin-like (CML) proteins, are triggered by PAMPs or effectors and subsequently activate complex downstream responses^38^. The CNGC has been suggested as an important Ca^2+^ conducting channel^39^. Studies have reported that group IV *CNGCs* regulate various resistance responses in tomato, likely by affecting Ca^2+^ signaling^40^. In the present study, the expression of three *CNGCs* and most of the genes related to calcium sensor proteins, such as *CDPKs*, *CaM* and *CML*, were inhibited. These results indicate that the Ca^2+^ signaling and signal transduction mediated through calcium sensor proteins were weakened in tomato root after DADS application. It has been reported that Ca^2+^ activates RBOH proteins, which are involved in the production of ROS *in vitro*^41^. In the present study, two of three *RBOH* genes were down-regulated, suggesting that DADS might affect the production of ROS. In contrast, a nucleotide binding site-leucine rich repeat protein (*RPS2*) gene and five *PR* genes were up-regulated during DADS treatment. It has been reported that RPS2 is a typical nucleotide-binding leucine-rich repeat resistance protein^42^, suggesting that DADS may induce plant defense responses through the activation of the plant ETI stage.

### Plant hormone synthesis and signal transduction in response to DADS treatment

Plant growth, development and responses to external conditions are regulated through phytohormones and their signal transduction. In the present study, the RNA-seq data indicated that the expression of DEGs associated with phytohormone signal transduction was opposite that of the corresponding DEGs associated with phytohormone synthesis (Fig. 5 and Supplementary Table S7). For example, the expression of the genes implicated in auxin, ABA and ethylene synthesis in tomato root was commonly down-regulated after exposure to DADS, while the genes related to auxin-, ABA- and ethylene-meditated signaling primarily showed up-regulated expression. These results suggest that phytohormone synthesis and signal transduction have coordinated and feedback regulation to respond to DADS.

In phytohormone signal transduction, *SAUR* and *GH3* are early auxin response genes^43^,^44^. *SAUR* is involved in tissue elongation and negatively influences the synthesis and polar transport of auxin^44^. The *GH3* genes encode a group of enzymes that adenylate IAA or conjugate free IAA with amino acids, thereby inhibiting plant growth. In the present study, the elevated expression of *SAURs* and decreased expression of *GH3* suggest that DADS promotes auxin content, consistent with a previous study^19^. In addition, auxin could induce the production of H_2_S, which promotes lateral root formation in tomato^45^. This implies that DADS may regulate tomato root development through multiple pathways that regulate auxin signal transduction. Previous studies have shown that salt stress increased IAA content in tomato seedlings, and IAA enhanced tomato tolerance to abiotic stress by activating the defense system^46^,^47^. Thus, DADS may promote the activities of defense enzymes by increasing the endogenous IAA content.

The plant hormone ABA plays a key role in regulating seed germination, plant development and stress responses^48^. In addition, SA-mediated pathogen responses are also negatively regulated through ABA^49^. In ABA synthesis, the overexpression of the rate-limiting enzyme NCED increased the ABA content and bacterial growth^50^. In the present study, the down-regulation of *NCED* suggested that DADS may inhibit the synthesis of ABA and induce the SA-mediated pathogen responses in tomato root. In ABA signal transduction, ABA binds to the PYR/PYL/RCAR family of receptors, followed by the deactivation of PP2C and the activation of SnRK2-type kinases, which contribute to the activation of ion channels in guard cells, leading to stomatal closure^48^. In the present study, we detected increased expression of *PYR*/*PYL* and *SnRK2* and decreased expression of *PP2C*, suggesting that DADS can positively regulate ABA signal transduction in tomato root thus affecting tomato responses to stimulation. In a previous study, the ABA content was increased in tomato plants under heat and salt stress^51^. The enhanced biosynthesis and signaling of ABA increased H_2_O_2_ production, thereby enhancing tomato tolerance against abiotic stress^52^,^53^. However, in the present study, DADS may act as a special signal that inhibits ABA synthesis but promotes ABA signal transduction.

Ethylene is a gaseous plant hormone implicated in plant developmental processes, such as root initiation and elongation, hypocotyl elongation, abscission, fruit ripening and senescence^54^. Previous studies have shown that exogenous ethylene application inhibits hypocotyl and root elongation and induces the radial swelling of hypocotyl and root cells in *Arabidopsis* seedlings^55^. In the present study, the expression of five *ACS* genes and one *ACO* gene was repressed after DADS treatment. *ACS* and *ACO* are involved in ethylene synthesis, and the down-regulation of these genes leads to reduced ethylene accumulation^56^. Interestingly, the dry weight of tomato root and aboveground parts and the length of the tomato root and shoot were all increased by DADS treatment (Supplementary Fig. S4). These results suggest that DADS may promote tomato growth by negatively regulating ethylene synthesis.

The regulation among different plant hormones and their homeostasis are important factors for plant development and interactions with the environment^57^. IAA, CTK, GA, ABA, and ethylene have all been implicated in DADS responses. However, the network of plant hormone signal transduction under DADS application requires further research.

## Conclusions

In the present study, dynamic analysis of the gene expression changes in DADS-treated tomato roots was performed using RNA-seq. A total of more than 2500 DADS-responsive genes were identified, including those involved in sulfur assimilation, GSH synthesis and metabolism, oxidation resistance, phytohormone synthesis and signal transduction, and those associated with plant-pathogen interactions. Defensive enzymes were also promoted after DADS treatment. DADS may activate tomato resistance in three ways: increasing the contents of nonenzymatic antioxidants, such as GSH; inducing the activity of enzymatic antioxidants, including SOD, POD, CAT, PPO and GR; and activating ETI. In addition, DADS may change the synthesis of plant hormones and signal transduction, thereby influencing tomato plant growth and resistance. This research may provide a comprehensive understanding of tomato root genes in response to DADS treatment. The results here also present valuable genetic resources for studies concerning the effects of DADS on plants and will provide new insight into genomic research on this topic.

## Materials and Methods

### Plant Materials and Chemicals

Tomato seeds (*Solanum lycopersicum* L. cv. Dongfen No. 3) were obtained from the Yufeng Seed Company (Yangling, China). Diallyl disulfide (DADS) was purchased from Fluka Chemika Co. (Bucha, Switzerland). DADS and Tween-80 were dissolved at a ratio of 1:2 (v:w) and stored at 4 °C after diluting to 20.67 mM as the stock solution.

### Culture Conditions and Treatments

This experiment was conducted in a growth chamber (RXZ 500D, Ningbo Jiangnan Instrument Factory, China), at 26/18 °C (day/night), 70% relative humidity and white light with an illumination of 30000 Lux during the day. Tomato seeds were planted in sterilized pearlite after disinfection. After tomato cotyledon expansion, tomato seedlings were cultivated with the half-strength Hoagland’s nutrient solution. At the 3-leaf stage, 50 mL of 0.21 mM DADS solution (containing 1X Hoagland’s nutrient solution) was added to each plant. The tomato roots were harvested after 0, 4, 24 or 48 h of DADS treatment and subsequently frozen in liquid nitrogen and stored at −80 °C prior to RNA extraction. This research was performed with three biological replications.

### Determination of physiological indices

The activity of Cys synthase was examined according to Gaitonde, and the production of Cys (nM) in one minute per gram fresh weight (Fw) was defined as a unit (U) of enzyme activity^58^. All other physiological indices, including the activities of SOD, POD, CAT and PPO, soluble protein content and MDA content after DADS treatment for 24 and 48 h, Fw and dry weight (Dw) of root and aboveground parts, and the root and shoot length after DADS treatment for 11 d, were determined as previously described^3^. SOD activity was determined as 50% inhibition of nitroblue tetrazolium reduction resulting from a photoreduced reaction. POD activity was measured using the guaiacol oxidation method. CAT activity was determined using an ultraviolet spectrophotometer to measure the decomposition of H_2_O_2_. PPO activity was measured using the catechol method. The MDA content was determined based on a thiobarbituric acid reaction and expressed as mM per gram Fw. The soluble protein content was determined through Coomassie brilliant blue G-250 staining and expressed as mg proteins per gram Fw. All indices were performed with three replications. The data were expressed as the means ± standard error (SE) and analyzed using Student's t-test (P<0.05).

### RNA extraction, library construction and sequencing

Twelve tomato root samples, including three biological replicates of a single plant per replicate, were used for RNA sequencing. Total RNA from each sample was isolated using Column Plant RNAOUT (Tiandz, Beijing). Subsequently, the RNA was treated with RNase-free DNase I (Promega, Madison, WI, USA) for 30 min at 37 °C to remove residual DNA. The quality and concentration of RNA were verified using a NanoDrop^TM^ 2000 UV-vis Spectrophotometer (Thermo Scientific, Waltham, MA, USA). An Agilent 2100 Bio-analyzer (Agilent Technologies, Santa Clara, CA) and RNase-free agarose gel electrophoresis were also used to qualify and quantify the 12 samples. Subsequently, the mRNA was enriched using oligo(dT) magnetic beads (Qiagen) and broken into short fragments. First-strand cDNA was synthesized using a random hexamer primer. Second-strand cDNA was subsequently generated using RNase H and DNA polymerase I. After purification, end reparation and poly (A) addition, sequencing adapters were ligated to the cDNA. Then the cDNA was purified through agarose gel electrophoresis and enriched by PCR amplification to generate the final cDNA library. Next, the cDNA libraries were sequenced on the Illumina HiSeq™ 2000 platform using the paired-end technology of Gene Denovo Co. (Guangzhou, China). Clean reads were selected by removing low quality reads, adaptor containing reads, and reads containing >5% N bases.

### DEGs and function enrichment analyses

Clean RNA-seq reads were mapped to the tomato reference genome. The normalized transcript abundance of the genes was calculated using the FPKM method, and subsequently, differential expression analysis was conducted using edgeR^59^. The false discovery rate (FDR) was used to determine the threshold of P value in multiple tests. In the present study, a threshold of FDR ≤ 0.05 and log_2_|FC (ratio of DADS/control)| ≥ 1 were used to determine the significant differences in gene expression. The DEGs were used for GO and KEGG enrichment analyses according to a previous study^60^. In the present study, both GO terms and KEGG pathways with Q-values ≤0.05 were considered significantly enriched in DEGs. The heat map was plotted using the OmicShare tools (www.omicshare.com/tools), a free online platform for data analysis.

### qRT-PCR confirmation of the RNA-seq data

The reliability and repeatability of the RNA-seq data were verified through qRT-PCR. The extraction and purification of RNA were performed as described above. The cDNA used for qRT-PCR was synthesized using the RevertAidTM Kit (Fermentas). Seventeen transcripts were randomly selected for qRT-PCR validation. Eight other genes involved in sulfur assimilation and GSH metabolism were also selected to determine the influence of DADS. The primers for the selected genes are listed in Supplementary Table S8. To normalize the total amount of cDNA present in each reaction, tomato *Actin-2*/*7* and *ubi3* were used as endogenous controls for calibration of the relative expression. The qRT-PCR was conducted with Maxima SYBR Green Master Mix (Thermo Scientific) using a Real-time Quantitative PCR System (iQ5, Bio-Rad, USA) with three repetitions. The relative expression data were analyzed using the 2^−ΔΔCT^ method.

### Availability of Data and Materials

The RNA-seq datasets generated from DADS- treated tomato roots are available in the NCBI Short Read Archive under accession number SRA398778 (http://www.ncbi.nlm.nih.gov/sra).

## Additional Information

**How to cite this article**: Cheng, F. *et al*. Transcriptomic insights into the allelopathic effects of the garlic allelochemical diallyl disulfide on tomato roots. *Sci. Rep.* **6**, 38902; doi: 10.1038/srep38902 (2016).

**Publisher's note:** Springer Nature remains neutral with regard to jurisdictional claims in published maps and institutional affiliations.

## Supplementary Material

Supplementary Information

Supplementary Table S3

Supplementary Table S4

## Figures and Tables

**Table 1:** Significantly enriched pathways involving DEGs following DADS treatment.

	Pathway	DEGs with pathway annotation (%)	All genes with pathway annotation (%)	Q-value	Pathway ID
Le4h vs. Le0h	Glutathione metabolism	28 (8.56%)	101 (2.3%)	3.68E-08	ko00480
	Plant hormone signal transduction	43 (13.25%)	262 (5.97%)	1.66E-05	ko04075
	Plant-pathogen interaction	30 (9.17%)	155 (3.53%)	2.23E-05	ko04626
	Cysteine and methionine metabolism	16 (4.89%)	83 (1.89%)	7.39E-03	ko00270
Le24h vs. Le0h	Plant hormone signal transduction	38 (19.91%)	262 (5.97%)	9.27E-10	ko04075
	Plant-pathogen interaction	26 (13.61%)	155 (3.53%)	4.98E-08	ko04626
Le48h vs. Le0h	Plant-pathogen interaction	21 (13.04%)	155 (3.53%)	7.89E-06	ko04626
	Plant hormone signal transduction	25 (15.53%)	262 (5.97%)	2.06E-04	ko04075

KEGG pathways with Q-values ≤0.05 were considered significantly enriched in DEGs.
