# Corrigendum: Transcriptomic insights into the allelopathic effects of the garlic allelochemical diallyl disulfide on tomato roots

**DOI:** 10.1038/srep42153

**Published:** 2017-02-09

**Authors:** Fang Cheng, Zhi-Hui Cheng, Huan-Wen Meng

Scientific Reports 6: Article number: 3890210.1038/srep38902; published online: 12 12 2016; updated: 02 09 2017

This article contains a typographical error in the legend of Figure 1 and in the Methods section under the subheading ‘DEGs and function enrichment analyses’, where

“log_2_|FC (ratio of DADS/control)| ≥ 1”

should read:

“|log_2_FC (ratio of DADS/control)| ≥ 1”.

