# Supplementary material for: Transcriptomic insights into the allelopathic effects of the garlic allelochemical diallyl disulfide on tomato roots
Source: Sci Rep. 2016 Dec 12;6:38902. doi: 10.1038/srep38902 (PMC5150855; doi:10.1038/srep38902)
Supplement: Supplementary Information [file srep38902-s1.pdf]

Transcriptomic insights into the allelopathic effects of the garlic allelochemical diallyl disulfide on tomato roots

Fang Cheng[1], Zhi-Hui Cheng*[1], Huan-Wen Meng[1]

***Correspondence:** Zhihui Cheng, College of Horticulture, Northwest A&F University, Taicheng Road No.3, Yangling, Shaanxi, 712100, China, chengzh@nwsuaf.edu.cn

Fang Cheng, College of Horticulture, Northwest A&F University, Yangling, Shaanxi 712100 China, chengfang@nwsuaf.edu.cn

Huanwen Meng, College of Horticulture, Northwest A&F University, Yangling, Shaanxi 712100 China, menghw2005@163.com

Supplementary Figure legends

Supplementary Figure S1. Sequencing quality assessment

Supplementary Figure S2. Sequencing saturation analysis. X-axis was number of clean reads; Y-axis was the percentage of identified genes.

Supplementary Figure S3. Correlations of expression value between replicates. X-axis and Y-axis was gene expression value of two replicates, respectively.

Supplementary Figure S4. The effects of DADS on the fresh weight and dry weight of tomato root and aboveground parts, and the length of shoot and root. Tomato plants at 3-leaf stage were treated with DADS (0.21 mM) and these physiological indexes were tested after 11 d of DADS application. Data for particular treatment are presented as mean ± SE. *Significant differences according to the Student's t-test ($P<0.05$), n = 3.

Supplementary Table legends

Supplementary Table S1. Throughput and quality of RNA-seq of the DEGs libraries

Supplementary Table S2. Mapping of the DEGs after removing rRNA

Supplementary Table S3. The summary results of different expressed genes and related information for all examples

Supplementary Table S4. GO classification of DEGs

Supplementary Table S5. The expression of DGEs relating to ROS scavenging

Supplementary Table S6. The expression of DEGs involved in plant-pathogen interaction

Supplementary Table S7. The expression of DEGs involved the biosynthesis of auxin, CTK, GA, ABA and ethylene

Supplementary Table S8. Primers for qRT-PCR

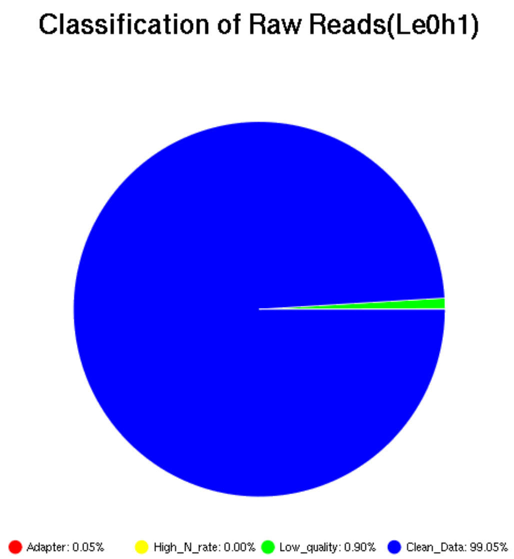
Classification of Raw Reads(Le0h1)
Adapter: 0.05%
High_N_rate: 0.00%
Low_quality: 0.90%
Clean_Data: 99.05%

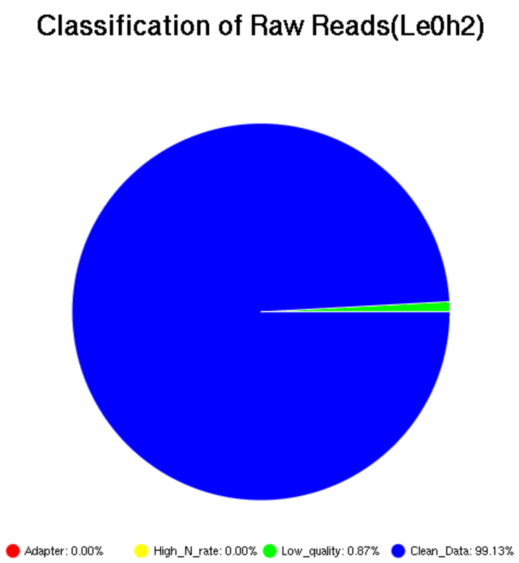
Classification of Raw Reads(Le0h2)
Adapter: 0.00%
High_N_rate: 0.00%
Low_quality: 0.87%
Clean_Data: 99.13%

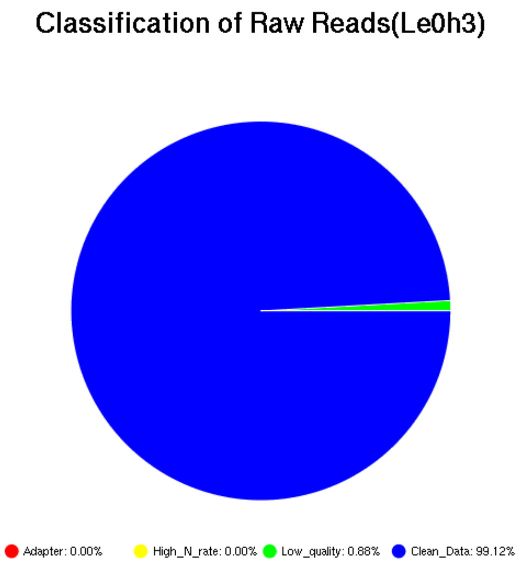
Classification of Raw Reads(Le0h3)
Adapter: 0.00%
High_N_rate: 0.00%
Low_quality: 0.88%
Clean_Data: 99.12%

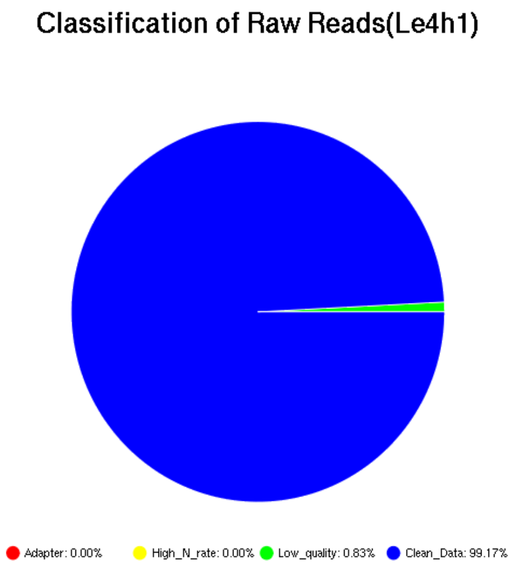
Classification of Raw Reads(Le4h1)
Adapter: 0.00%
High_N_rate: 0.00%
Low_quality: 0.83%
Clean_Data: 99.17%

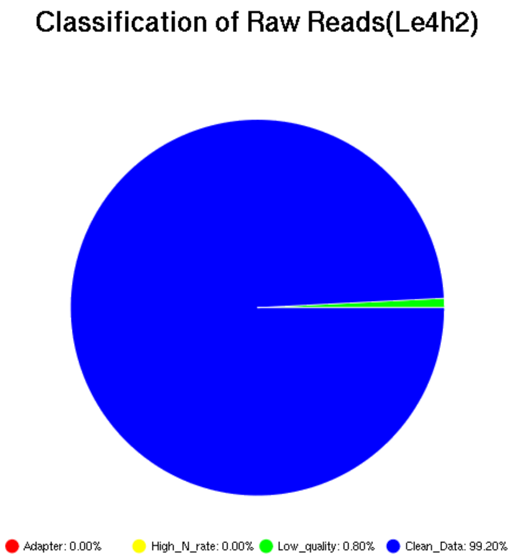
Classification of Raw Reads(Le4h2)
Adapter: 0.00%
High_N_rate: 0.00%
Low_quality: 0.80%
Clean_Data: 99.20%

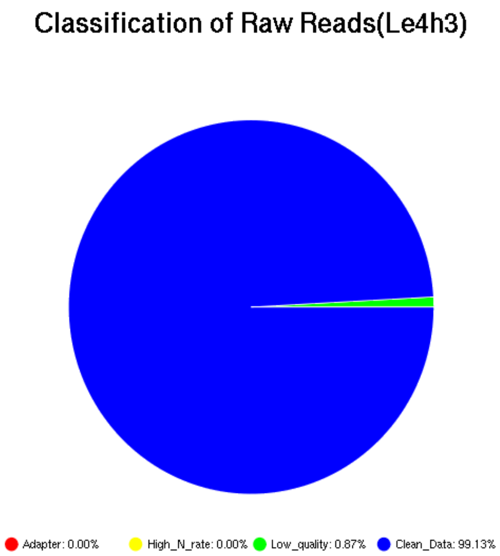
Classification of Raw Reads(Le4h3)
Adapter: 0.00%
High_N_rate: 0.00%
Low_quality: 0.87%
Clean_Data: 99.13%

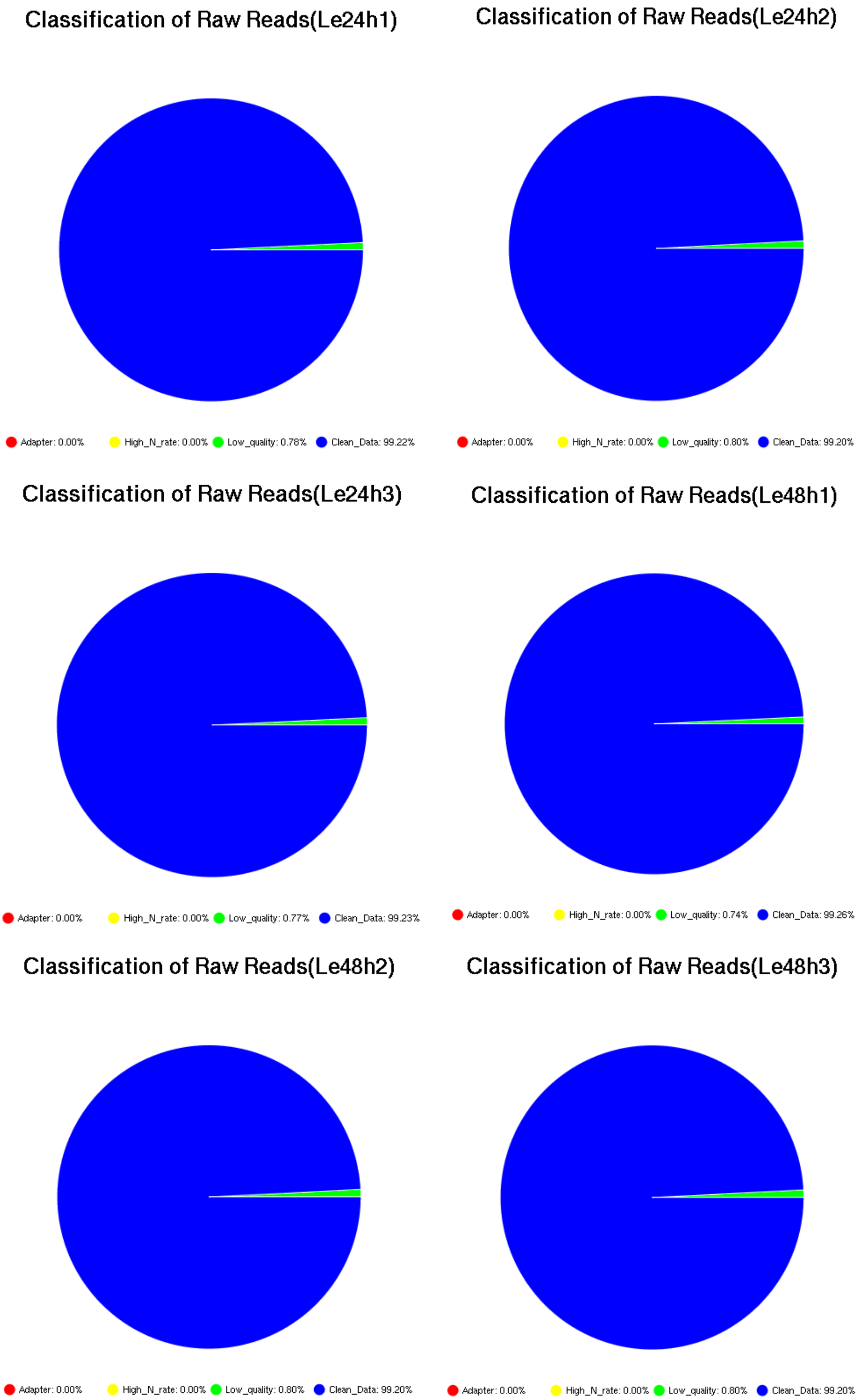

Supplementary Figure S1. Sequencing quality assessment

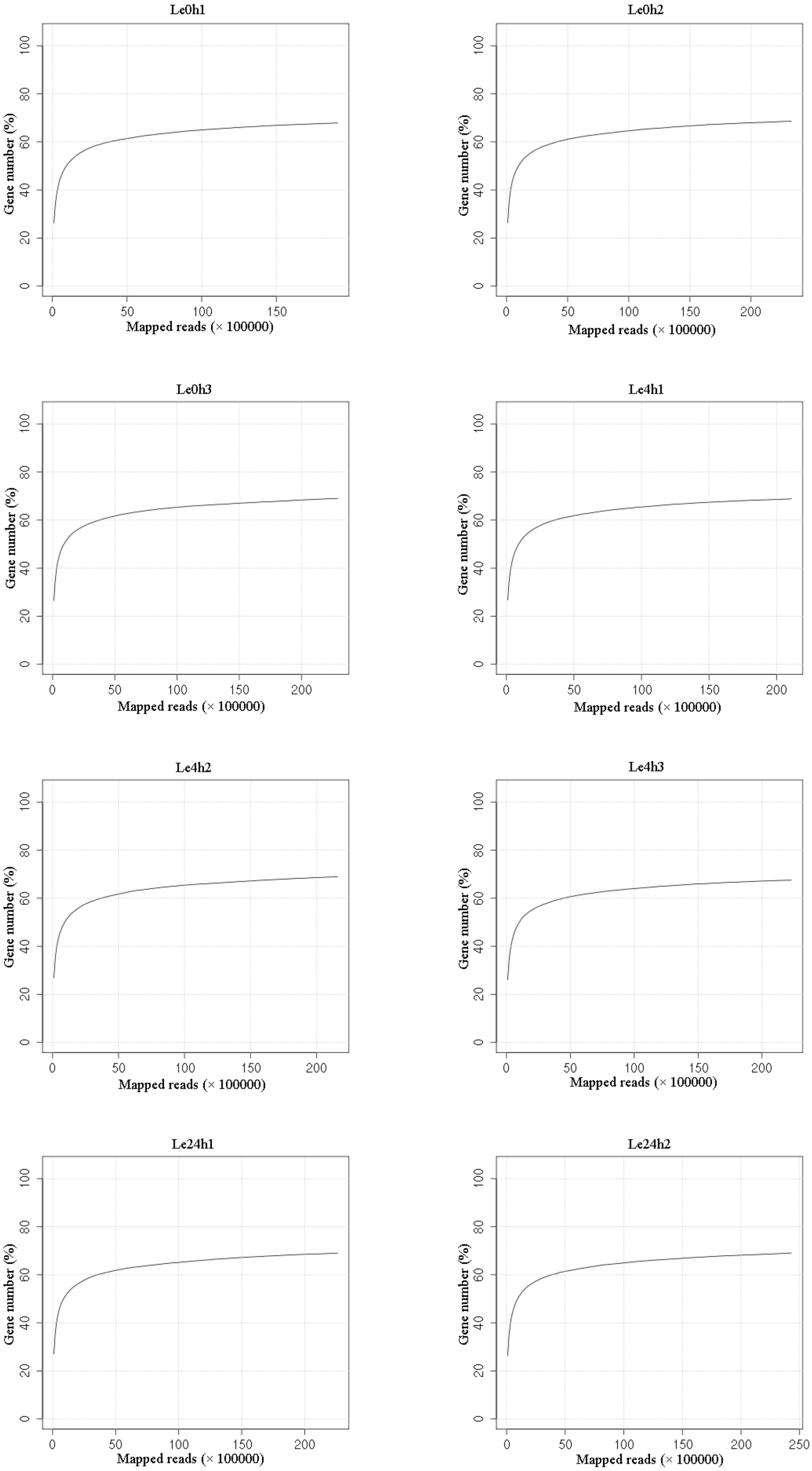
Le0h1
Le0h2
Le0h3
Le4h1
Le4h2
Le4h3
Le24h1
Le24h2
Gene number (%)
Mapped reads (× 100000)

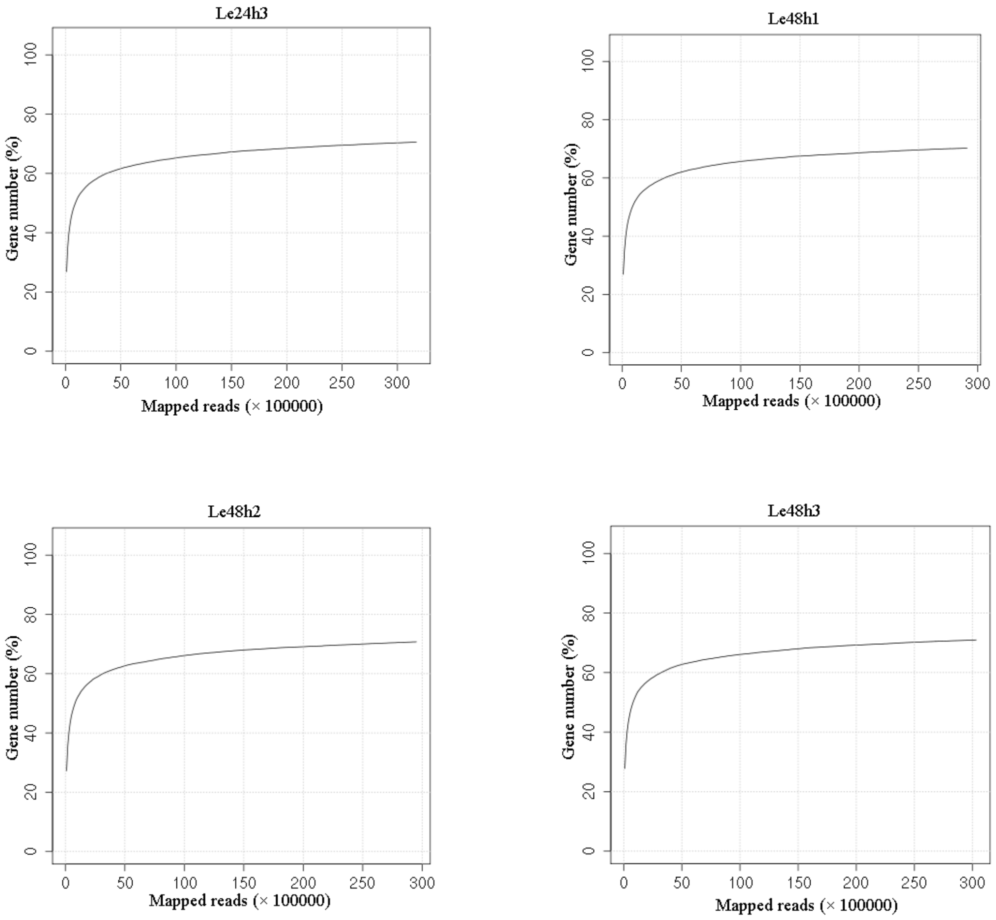

Supplementary Figure S2. Sequencing saturation analysis. X-axis was number of clean reads; Y-axis was the percentage of identified genes.

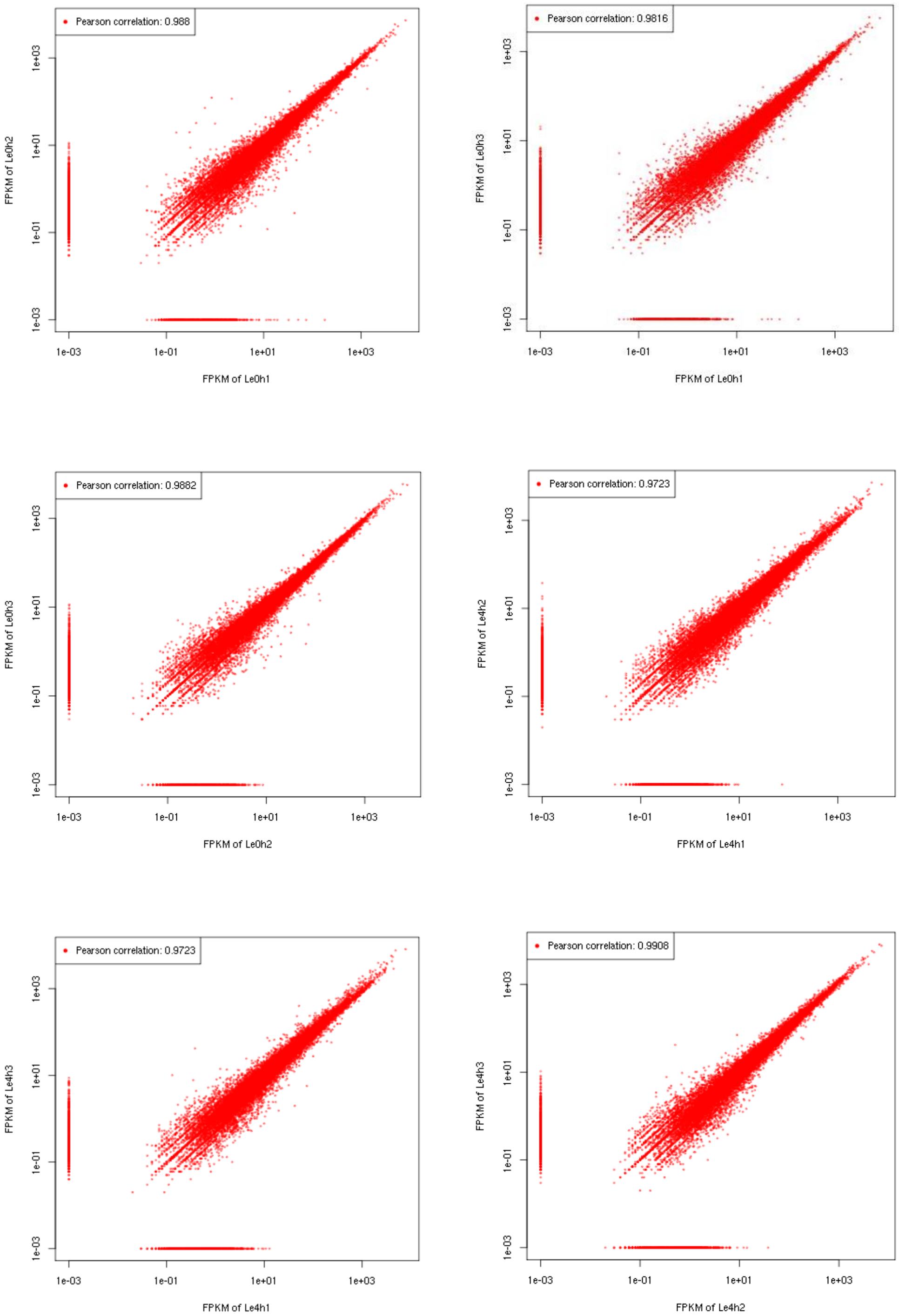

Pearson correlation: 0.988
FPKM of Le0h2
FPKM of Le0h1
Pearson correlation: 0.9816
FPKM of Le0h3
FPKM of Le0h1
Pearson correlation: 0.9882
FPKM of Le0h3
FPKM of Le0h2
Pearson correlation: 0.9723
FPKM of Le4h2
FPKM of Le4h1
Pearson correlation: 0.9723
FPKM of Le4h3
FPKM of Le4h1
Pearson correlation: 0.9908
FPKM of Le4h3
FPKM of Le4h2
1e-03
1e-01
1e+01
1e+03

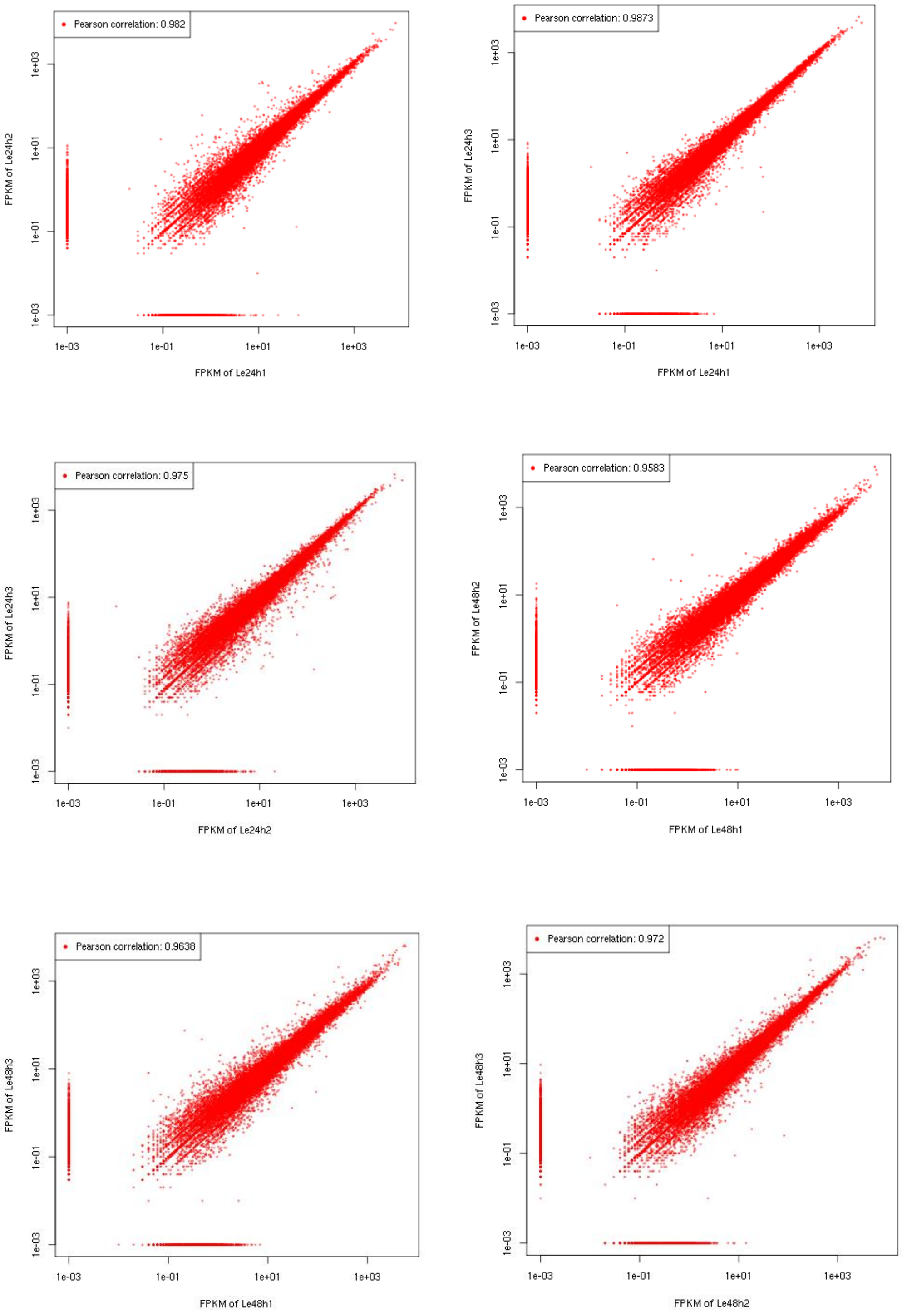

Supplementary Figure S3. Correlations of expression value between replicates. X-axis and Y-axis was gene expression value of two replicates, respectively.

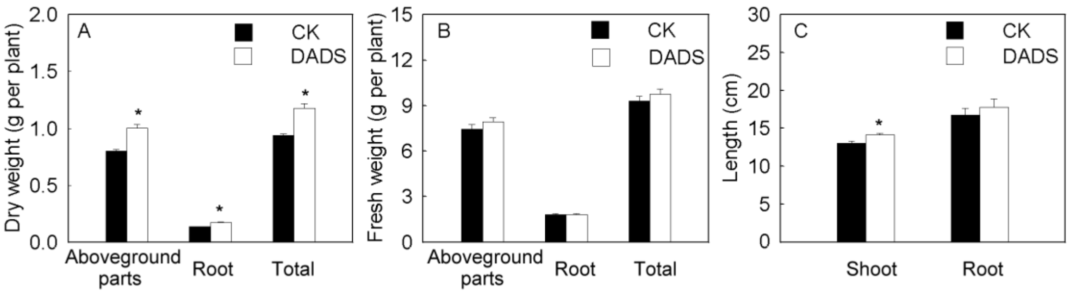

Supplementary Figure S4. The effects of DADS on the fresh weight and dry weight of tomato root and aboveground parts, and the length of shoot and root. Tomato plants at 3-leaf stage were treated with DADS (0.21 mM) and these physiological indexes were tested after 11 d of DADS application. Data for particular treatment are presented as mean ± SE. *Significant differences according to the Student's t-test ($P<0.05$), $n = 3$.

Supplementary Table S1. Throughput and quality of RNA-seq of the DEGs libraries

Sample	The bases information After Filter			The reads information after filter		
	HQ Clean Data (bp)	GC (%)	Q30 (%)	HQ Clean Reads Num (%)	GC (%)	Q30 (%)
Le0h1	2081735800 (99.05 %)	45.35	93.71	20817358 (99.05%)	45.34	94.14
Le0h2	2156161200 (99.13 %)	43.71	94.30	21561612 (99.13%)	43.70	94.71
Le0h3	2115051800 (99.12 %)	43.65	94.28	21150518 (99.12%)	43.64	94.70
Le4h1	1960414000 (99.17 %)	43.79	94.44	19604140 (99.17%)	43.78	94.83
Le4h2	1998753400 (99.20 %)	43.84	94.47	19987534 (99.2%)	43.83	94.85
Le4h3	2066345600 (99.13 %)	43.70	94.38	20663456 (99.13%)	43.69	94.79
Le24h1	2084544800 (99.22 %)	43.65	94.63	20845448 (99.22%)	43.64	95.01
Le24h2	2265605000 (99.20 %)	43.88	94.58	22656050 (99.2%)	43.88	94.97
Le24h3	2926257600 (99.23 %)	43.47	94.76	29262576 (99.23%)	43.47	95.14
Le48h1	2681345400 (99.26 %)	43.55	94.82	26813454 (99.26%)	43.55	95.19
Le48h2	2733395800 (99.20 %)	43.45	94.58	27333958 (99.2%)	43.45	94.96
Le48h3	2792807000 (99.20 %)	43.62	94.67	27928070 (99.2%)	43.61	95.05

Supplementary Table S2. Mapping of the DEGs after removing rRNA

Sample	Total Reads	Unique Mapped Reads	Multiple Mapped reads	Mapping Ratio
Le0h1	17603426	15629371 (88.79%)	118430 (0.67%)	89.46%
Le0h2	21447790	19347190 (90.21%)	161740 (0.75%)	90.96%
Le0h3	21066430	18926417 (89.84%)	156624 (0.74%)	90.59%
Le4h1	19443330	17426104 (89.63%)	121902 (0.63%)	90.25%
Le4h2	19859412	17890579 (90.09%)	139910 (0.70%)	90.79%
Le4h3	20583316	18573094 (90.23%)	152280 (0.74%)	90.97%
Le24h1	20816946	18894573 (90.77%)	136702 (0.66%)	91.42%
Le24h2	22458502	20137332 (89.66%)	144502 (0.64%)	90.31%
Le24h3	29222924	26524352 (90.77%)	180530 (0.62%)	91.38%
Le48h1	26781196	24683564 (92.17%)	175900 (0.66%)	92.82%
Le48h2	27297770	24633500 (90.24%)	201716 (0.74%)	90.98%
Le48h3	27890586	25524745 (91.52%)	197612 (0.71%)	92.23%

Supplementary Table S5. The expression of DGEs relating to ROS scavenging

Gene ID	Log_2(fold change)			Description
	4h vs. 0h	24h vs. 0h	48h vs. 0h	
Solyc02g090470.2	**3.53**	**1.37**	**2.01**	Peroxidase 10-like
Solyc01g099620.2	**2.20**	**1.24**	**2.44**	Peroxidase 24
Solyc02g090450.2	**2.50**	**1.53**	**1.48**	Peroxidase 10-like
Solyc01g067860.2	**1.08**	**0.53**	**0.58**	Peroxidase 19-like
Solyc05g055320.2	**0.95**	**0.50**	**1.07**	Peroxidase 5-like
Solyc03g120800.2	**0.40**	**0.00**	**1.36**	Peroxidase
Solyc02g083480.2	-0.16	0.68	1.15	Peroxidase 64-like
Solyc12g005790.1	-1.23	0.70	0.21	Peroxidase 27-like
Solyc05g052280.2	1.69	-0.08	0.61	Peroxidase 4-like
Solyc01g108320.2	1.61	0.30	-0.08	Peroxidase 10-like
Solyc02g084800.2	1.30	-0.29	0.06	Peroxidase
Solyc02g087070.2	2.18	-0.71	0.03	Alpha-DOX1
Solyc05g050870.2	1.26	-0.40	-0.10	Cationic peroxidase 1-like
Solyc05g050890.1	1.14	-0.29	-0.22	Peroxidase
Solyc01g105070.2	0.68	-1.22	-0.79	Cationic peroxidase
Solyc10g076190.1	-0.10	-1.59	0.01	Peroxidase 4-like
Solyc04g080330.2	-1.59	-0.05	-0.83	Peroxidase
Solyc06g068680.2	-1.50	-0.74	-0.84	Respiratory burst oxidase-like protein
Solyc06g075570.1	-1.27	-0.79	-0.48	Respiratory burst oxidase protein A
Solyc02g094180.2	-1.02	-1.71	-1.55	Peroxidase 51-like
Solyc01g067870.2	-1.75	-0.18	-0.25	peroxidase 19-like
Solyc01g101050.2	-1.83	-1.77	-1.01	Peroxidase 5-like
Solyc03g117980.2	-1.40	-2.32	-2.05	Whitefly-induced gp91-phox
Solyc11g018800.1	-1.21	-2.97	-1.54	Peroxidase 2
Solyc08g074630.1	-1.03	0.39	0.87	Polyphenol oxidase F

Supplementary Table S6. The expression of DEGs involved in plant-pathogen interaction

Gene ID	Log$_2$(fold change)			Description	Symbol
	4h vs. 0h	24h vs. 0h	48h vs. 0h		
Solyc02g083850.2	1.00	0.45	0.57	Calcium-dependent protein kinase	CDPK
Solyc03g033540.2	-1.08	-1.11	-0.96	Calcium-dependent protein kinase	CDPK
Solyc10g079130.1	-0.90	-1.11	-1.00	Calcium-dependent protein kinase 32-like	CDPK
Solyc03g117980.2	-1.40	-2.32	-2.05	Whitefly-induced gp91-phox	Rboh
Solyc06g068680.2	-1.50	-0.74	-0.84	Respiratory burst oxidase homolog protein D-like	Rboh
Solyc01g099620.2	2.20	1.24	2.44	Respiratory burst oxidase homolog B	Rboh
Solyc02g086990.2	-1.20	-0.34	-0.29	Putative cyclic nucleotide-gated ion channel 18-like	CNGCs
Solyc02g088560.2	-1.69	-0.59	-0.46	Cyclic nucleotide-gated ion channel 2-like	CNGCs
Solyc01g095770.2	-3.21	-3.08	-2.66	Cyclic nucleotide-gated ion channel 1-like	CNGCs
Solyc11g071760.1	-3.11	-5.27	-2.14	Regulator of gene silencing	CaM/CML
Solyc03g005040.1	-2.68	-13.44	-2.00	Putative calcium-binding protein CML19-like	CaM/CML
Solyc02g094000.1	-2.32	-5.26	-2.59	Putative calcium-binding protein CML19-like	CaM/CML
Solyc02g091500.1	-2.10	-2.84	-1.34	Calcium-binding protein CML24-like	CaM/CML
Solyc11g071740.1	-1.82	-4.38	-1.93	Calcium-binding protein CML38-like	CaM/CML
Solyc02g088090.1	-1.50	-3.17	-1.91	Probable calcium-binding protein CML30-like	CaM/CML
Solyc02g063350.1	-1.75	-2.08	-1.18	Probable calcium-binding protein CML23-like	CaM/CML
Solyc03g118810.1	-1.54	-2.66	-1.35	Calcium-binding allergen Ole e 8-like	CaM/CML
Solyc06g073830.1	-1.01	-2.85	-1.71	Calcium-binding protein CML38-like	CaM/CML
Solyc10g074740.1	-1.22	-2.17	-1.29	Calcium-binding protein CAST-like	CaM/CML
Solyc10g081170.1	-1.24	-1.02	-0.63	Calmodulin	CaM/CML
Solyc06g053930.2	-1.10	-0.94	-0.88	Calmodulin-like protein 8-like	CaM/CML
Solyc04g018110.1	-0.89	-1.47	-0.97	Hop-interacting protein THI026	CaM/CML
Solyc06g068960.1	-0.75	-1.64	-1.46	Calcium-binding allergen Ole e 8-like	CaM/CML

Solyc10g079420.1	-0.34	-1.08	-0.37	Probable calcium-binding protein CML36-like	CaM/CML
Solyc02g090810.2	1.12	0.09	0.61	Caltractin-like	CaM/CML
Solyc09g014990.2	-2.50	-5.00	-3.31	Probable WRKY transcription factor 33-like	WRKY25/33
Solyc06g066370.2	-1.73	-3.67	-2.11	Probable WRKY transcription factor 33-like	WRKY25/33
Solyc10g011910.2	-1.50	-3.56	-1.42	WRKY transcription factor 22-like	WRKY22/29
Solyc02g077370.1	0.10	-2.87	-1.43	PTI5	PTI5
Solyc06g082590.1	-1.07	-2.29	-1.01	PTI6	PTI6
Solyc08g080670.1	3.25	1.34	1.43	Pathogenesis-related 5-like protein	PR
Solyc08g079900.1	1.98	1.77	1.50	Pathogenesis related protein P69G	PR
Solyc10g076440.1	1.18	1.23	0.96	Nucleotide binding site-leucine rich repeat protein	PR
Solyc04g064870.2	2.52	0.02	0.88	Pathogenesis-related protein-like protein	PR
Solyc07g006700.1	-0.23	1.37	1.19	pathogenesis-related protein PR-1-like	PR
Solyc08g068990.1	0.36	1.76	0.92	Pathogenesis-related protein 1	PR
Solyc09g090970.2	1.03	-0.18	0.34	Similar to pathogenesis-related protein STH-2	PR
Solyc01g097240.2	0.18	-1.45	0.21	Pathogenesis-related protein P2	PR
Solyc01g106620.2	1.75	-0.57	-1.14	PR1 protein	PR
Solyc10g076440.1	1.18	1.23	0.96	Nucleotide binding site-leucine rich repeat protein	RPS2
Solyc12g009220.1	-4.06	-5.59	-3.70	Jasmonate ZIM-domain protein 1	JAZ
Solyc03g122190.2	-4.19	-4.34	-3.45	salt responsive protein 1	JAZ
Solyc07g042170.2	-1.81	-2.50	-1.18	protein TIFY 10A-like	JAZ
Solyc08g076930.1	-1.23	-2.46	-1.37	jasmonic acid 3	MYC2

Supplementary Table S7. The expression of DEGs involved the biosynthesis of auxin, CTK, GA, ABA and ethylene

Gene ID	$\log_2$(fold_change)			Description
	4h vs. 0h	24h vs. 0h	48h vs. 0h	
**Auxin**				
Solyc02g084640.2	-1.18	-0.39	-0.31	Aldehyde dehydrogenase
Solyc09g074430.2	-1.99	-0.10	-0.08	Flavin-containing monooxygenase YUCCA10-like
**CTK**				
Solyc09g064910.1	1.38	1.50	0.95	Adenylate isopentenyltransferase
Solyc04g016430.2	0.36	0.59	1.32	Cytokinin oxidase/dehydrogenase-like 5
Solyc04g080820.2	1.18	0.27	0.03	Cytokinin oxidase/dehydrogenase-like
Solyc01g088160.2	-1.64	-2.09	-1.10	Cytokinin oxidase/dehydrogenase-like 2
Solyc07g006800.1	2.47	0.61	1.19	Zeatin O-glucosyltransferase-like
Solyc04g008330.1	-3.10	0.26	0.96	UDP-glucuronosyl/UDP-glucosyltransferase
Solyc10g079930.1	0.33	-1.78	-0.92	Zeatin O-glucosyltransferase-like
Solyc04g016230.2	2.40	0.37	1.06	Zeatin O-glucosyltransferase-like
Solyc05g012670.1	-3.94	-1.66	-0.56	Glucosyltransferase-3;UDP-glucuronosyl/UDP-glucosyltransferase
**GA**				
Solyc08g005640.2	-1.42	-0.26	0.25	Ent-kaurene synthase-like protein 1
Solyc11g072310.1	1.09	0.79	0.47	Gibberellin 20-oxidase-3
Solyc07g061720.2	0.88	1.27	2.61	Gibberellin 2-oxidase
Solyc01g079200.2	-2.48	-3.62	-2.66	Gibberellin 2-oxidase
Solyc02g070430.2	0.86	1.69	0.93	Gibberellin 2-beta-dioxygenase-like
Solyc06g066820.2	1.23	1.60	2.08	3b-hydroxylase
**ABA**				
Solyc01g005940.2	-0.36	-4.73	-3.31	Phytoene synthase 3
Solyc03g031860.2	-1.64	-0.68	-0.11	Phytoene synthase 1

Solyc02g081330.2	-1.27	-0.64	-0.29	Phytoene synthase 2
Solyc03g007960.2	-1.93	-2.08	-0.56	Beta-carotene hydroxylase
Solyc06g036260.2	-0.72	0.17	0.56	Beta-carotene hydroxylase 1
Solyc07g056570.1	**-1.97**	**-2.81**	-0.82	Nine-cis-epoxycarotenoid dioxygenase
Solyc11g071600.1	0.89	-0.38	-0.02	Aldehyde oxidase; Aldehyde oxidase TAO3
Solyc01g108210.2	-2.40	-1.47	-1.22	Cytochrome P450
Solyc08g005610.2	-0.31	-2.12	-0.85	ABA 8'-hydroxylase CYP707A2
Solyc04g078900.2	-9.13	-2.69	-1.58	ABA 8'-hydroxylase
**Ethylene**				
Solyc03g043890.2	-1.83	-0.83	-0.35	1-aminocyclopropane-1-carboxylate synthase
Solyc08g081550.2	-1.29	-1.63	-0.70	1-aminocyclopropane-1-carboxylate synthase
Solyc12g056180.1	-2.33	-1.55	-2.62	1-aminocyclopropane-1-carboxylate synthase
Solyc08g081540.2	-1.14	-1.42	-0.77	1-amino-cyclopropane-1-carboxylate synthase
Solyc02g091990.2	-2.63	-4.08	-1.77	1-aminocyclopropane-1-carboxylate synthase 3
Solyc01g095080.2	0.99	-1.23	0.31	1-aminocyclopropane-1-carboxylate synthase 2
Solyc07g026650.2	6.06	5.77	6.51	1-aminocyclopropane-1-carboxylate oxidase
Solyc02g036350.2	-0.35	-0.99	-1.44	1-aminocyclopropane-1-carboxylate oxidase
Solyc07g049550.2	2.63	0.24	1.17	1-aminocyclopropane-1-carboxylate oxidase 4
Solyc07g049530.2	1.03	-1.37	0.15	1-aminocyclopropane-1-carboxylate oxidase 1

Supplementary Table S8. Primers for qRT-PCR

	Unigene ID	Description	Forward primer (5'→3')	Reverse primer (5'→3')
1	Solyc07g024000.2	*chlorophyll(ide) b reductase*	ACCGACCTACTTCTGAGTGGA	CCTGATCATCAAACCATCGGC
2	Solyc06g074990.1	*NRT2;1*	GGGTGCAATTCCGTTCACTGCTT	GCAGCATACCACAATATGTTACCG
3	Solyc11g013810.1	*NR*	AACGCGGAAGCTTGGTGGTA	AACCTCGAGTGACCAAAAGCAC
4	Solyc04g080820.2	*CKX4*	TCGCACCGAAATCATGGACT	CGAATCCACCTCACCCTTTG
5	Solyc02g090450.2	*peroxidase 10*	TGCTGCACTGGTGTCAAACTA	TGGAACTTTCTAATTCACCACCC
6	Solyc08g082670.2	*cellulose synthase*	GCATTGTACATTCTATAAGACCTCC	AGGAAGAATGCCTACAAGGGTC
7	Solyc07g052980.2	*XTH16*	TGGTGGGTTTGGTTAGCTCTGC	CCCAGTACCAGAAAGATGATCC
8	Solyc07g056480.2	*S-transferase/peroxidase*	GGCTAAGGATTGCACTTGCTG	CCTCCCAGAATCATACAACTTCTT
9	Solyc01g068410.2	*PIN5*	CGTAGCTAGCAGGTGGCATT	AAGTGCCATTGTTGCTGGAC
10	Solyc09g015830.1	*receptor-like protein kinase FERONIA*	CCTCATCTTTAAGTGATTTCTGCG	ATCTGTGCCCCATTTCCGAC
11	Solyc09g065850.2	*IAA3*	TTGCCTGGGATAATAAACGATG	AACAACTTGTGCTTTTGGTGC
12	Solyc07g042550.2	*sus3*	TCCTCCATCCATTACTTCCCTCTAT	TTTCGATCCTTGAAAGAAAGAGCA
13	Solyc06g005170.2	*MPK3*	TGGAATCGTCTGCTCTGTGTT	AAGGGCGGAGGAATCACATC
14	Solyc01g107730.2	*CycD3;2*	GGGAGTGACCCTTTTCTCTGT	GCATCCTCCACTTGGAAGTCA
15	Solyc01g079200.2	*GA2ox3*	ATTGGGGCCTATCACCAAATC	TCGTCAACGCCTGAAAGGAG
16	Solyc04g025880.2	*K transporter6*	GAACGTCGAAGCACACTACAC	ACTAGCTTCCATCAGTTCGCA
17	Solyc07g049550.2	*ACO4*	CCAGCTTGAGGTGATTACCAA	ACGTTTGTATAACTTCCAAGAACAT
18	Solyc03g005260.2	*APS1*	TCCCACTGCCATTTTAAGCGAT	AGCAGGTGAAAGTCGGAACC
19	Solyc11g065620.1	*sir*	AAGTTGTGAAAGCTCGGAATGATAACT	TTCTCCATCCTCATCAGATACAACAAC
20	Solyc10g012370.2	*cysteine synthase 1*	GAGAGATAACAAGCTCGGCCA	CTTCTCCTCACCAACGACGC
21	Solyc01g097920.2	*cysteine synthase-like*	TCCCTGGATAAGGAAGGTAGCA	GAGGACAGACTTGCCAGGGG
22	Solyc07g065340.1	*SAT3*	AGCAAAATGCAACAAAATCCACAA	GTGGCTTGTTTGGGTCACGA
23	Solyc08g081010.2	*GSH1*	TCTAGCCGCCTCAGCACAC	GCCATGCTTTGTGCCTTGG

24	Solyc09g091840.2	*GR*	TGTGCTGTGTTCTGCATCCC	CTGCTCCTTGGTTGCTCCAC
25	Solyc00g187050.2	*Lap2*	TCGGAGCTGGTTCGAGGATT	GCATCAGCAAGTGTGAGCCT
reference gene 1	NM_001308447	*Actin-2/7*	GGACTCTGGTGATGGTGTTAG	CCGTTCAGCAGTAGTGGTG
reference gene 2	XM_004229002	*ubi3*	AGGTTGATGACACTGGAAAGGTT	ATCGCCTCCAGCCTTGTTGTA
